# Compounding a Felony!

**Published:** 1898-04-30

**Authors:** 


### The Hospital

A Journal of

The Medical Sciences and Hospital Administration.

Vol. XXIV.—No. 605.] Edited by Sir Henry Burdett, K.C.B., and Solomon C. Smith, M.D., M.R.C.P. [April 30, 1898.

### Compounding a Felony!

Medical men stand in such intimate relationship with the family life of the nation, they of necessity become the depositaries of such confidential communications, and the good that they are able to do depends so largely upon the maintenance of that seal of professional secrecy which, whatever may be said by some to the contrary, does as a fact protect the confessions of the consulting-room, that it becomes a matter of the supremest interest, to the public quite as much as to the profession, to protest at once against any attempt to turn the trusted medical adviser into a police agent and a public informer. Let us then in a few words tell a little history about a tragedy which happened only a week or two ago. A single lady, thirty-three years of age, became much depressed in mind and, when staying with a cousin, who was a medical man in London, she tried to commit suicide by taking laudanum. She then went to live with a medical man at Ewell as a voluntary boarder. While there she appeared to be suffering from hysteria and occasional depression, but she could manage her own affairs and was usually bright and cheerful. As a precaution she had a nurse, but she never made any further attempt to do herself any harm until one night she got out by the window and drowned herself. All this is very simple and very sad, and is but another illustration of the fact that there are plenty of people at large who are undoubtedly at times insane, and yet are not so insane as to come under the lunacy law as it exists at present.

Our present interest in the case, however, centres in the inquest. It was clearly desirable to get some account of the previous attempt at suicide as bearing upon her state of mind, and Dr. Kingsforde, of Brondesbury, volunteered to give evidence. He said he was cousin to the deceased, who, prior to going to Ewell, was a month in his house. It was at his house that deceased took poison. On his saying this, the coroner pointed out the position witness was in, and cautioned him that what he said might hereafter operate adversely against him. Later on he said, "I think it necessary to give you a warning. Compounding a felony is a very serious thing. If a person takes poison, that is *prima facie* evidence that they want to do themselves an injury, and that is an offence to our law . . . . I think it right to warn you that you need not answer any questions I put to you, but if you do I should be bound to take them down, and it will be my duty to bring the matter before the proper authorities." We rub our eyes! Compounding a felony because he did not report an attempt at suicide! Can it be really true that it is a doctor's duty, on his way home from his morning's round, to call in at the police-station and report any little information which he may have picked up as to possible crimes committed by his patients? The very suggestion is an outrage. Fortunately, however, the profession has the distinct ruling of an eminent judge as a guide to conduct in such cases, and the matter is so important, and it is so desirable that the proper relation between the medical profession and the public should be clearly understood, that we quote the following from the summing up by Mr. Justice Hawkins in the well-known case of Kitson v. Playfair and wife. His lordship is reported to have said:

"It was also said by the medical witnesses that, if in the course of professional practice they came across a case which indicated either that a crime had been committed or was about to be committed, that under those circumstances they were bound to divulge it. To whom? To the Public Prosecutor! If a poor, wretched woman committed an offence for the purpose of getting rid of that with which she was pregnant, and of saving her character, her reputation, and, it might be, her very means of livelihood, and if a doctor was called in to assist her—not in procuring abortion, for that in itself was a crime—but called in for the purpose of attending her and giving her medical advice—how she might be cured so as to go forth about her business—he (the learned Judge) doubted very, very, very much whether he would be justified in going forth and saying to the Public Prosecutor: 'I have been attending a poor young woman who has been trying to procure abortion with the assistance of her sister. She is now pretty well, and is getting better, and in the course of a few days she will be out again, but I think I ought to put you on to the woman.'

"To his (the learned Judge's) mind, a thing like that would be a monstrous cruelty. He did not know what the jury's views would be; he spoke only of his own. Therefore, when it was said that there was a general rule existing in the medical profession that whensoever they saw in the course of their medical attendance that a crime had been committed, or was about to be committed, they were in all cases to go off to the Public Prosecutor, he (the learned Judge) was bound to say that it was not a rule which met with his approbation, and he hoped it would not meet with the approbation of anybody else."

This seems perfectly plain, and a general acceptance of the principles so well laid down by Mr. Justice Hawkins is most desirable. The idea that, after stomach-pumping a poor wretch, and bringing her back to the life she wanted to escape from, a doctor is bound straightway to hale her before the magistrate, and so upset such chance of sanity as remains to her by the shame and terror of the police-court, is absurd.

